# Understanding Human Epidermal Stem Cells at Single-Cell Resolution

**DOI:** 10.1016/j.jid.2022.04.003

**Published:** 2022-08

**Authors:** Victor Augusti Negri, Fiona M. Watt

**Affiliations:** 1 Centre for Gene Therapy and Regenerative Medicine, Faculty of Life Sciences & Medicine, School of Basic & Medical Biosciences, King’s College London, London, United Kingdom; 2 Directors' Research Unit, European Molecular Biology Laboratory, Heidelberg, Germany

**Keywords:** EB, epidermolysis bullosa, ECM, extracellular matrix, ERK, extracellular signal‒regulated kinase, HCA, The Human Cell Atlas, IFE, interfollicular epidermis, K, keratin, scRNA-seq, single-cell RNA sequencing

## Abstract

The human epidermis is one of the first tissues in which the existence of stem cells was recognized and is one of the few in which ex vivo expansion for tissue repair is established clinically. Nevertheless, the nature of stem cells has been elusive. Using clonal growth assays of cultured keratinocytes as a quantitative measure of their abundance, several candidate stem cell markers have been described. Recently, the volume and quality of single-cell RNA-sequencing datasets have increased exponentially, providing new opportunities to explore the nature of epidermal stem cells and test the validity of in vitro experimental models.

**Editor's Note**

This Review is part of a series of articles invited from lectures presented at the 2021 Meeting of the European Society for Dermatological Research in celebration of its 50th Anniversary.

## Introduction

The human interfollicular epidermis (IFE) is one of the tissues in the human body with a high cell turnover rate. Throughout adulthood, stem cells that are attached to the basal layer self-renew and give rise to the differentiating cells of the suprabasal spinous and granular layers, before losing their nucleus and cytoplasmic organelles and forming cornified cells that are shed from the skin surface (Watt, 2014). Under steady-state (homeostatic) conditions, the rate of production of new cells in the basal layer is balanced by the rate of loss of cornified cells, and so the thickness of the epidermis remains constant (Leblond, 1964). However, in hyperproliferative conditions, such as psoriasis and eczema, proliferation is increased, and there is a corresponding increase in epidermal thickness (Pasparakis et al., 2014).

## In Vivo Evidence for Heterogeneity of Basal Layer Keratinocytes

Early studies in rodents established that the basal cells of the IFE differ from one another. In some regions of mouse IFE, the keratinocytes are arranged in columns underlying a single stack of cornified cells (Mackenzie, 1970). Basal cells at the center of each column divide less frequently than those at the periphery, leading to the concept of a central, slowly cycling stem cell that produces daughter―transit-amplifying―cells that undergo a few rounds of divisions before moving into the suprabasal layers and undergoing terminal differentiation (Jones et al., 2007; Mackenzie, 1970; Potten, 1975).

To label slowly cycling stem cells, neonatal mice can be subjected to long-term DNA labeling with tritiated thymidine or BrdU, the rationale being that as the skin is expanding in the first days after birth, both stem and transit-amplifying cells are dividing and will be labeled (Bickenbach, 1981; Braun and Watt, 2004). In adult mice, the transit-amplifying cells will continue to divide and will therefore lose the DNA label, whereas the stem cells will divide infrequently and therefore retain the label (label-retaining cells) (Bickenbach, 1981). Extensive studies have confirmed the existence of label-retaining cells within mouse epidermis, particularly within the hair follicle bulge (Cotsarelis et al., 1990).

An elegant strategy was subsequently developed that enables fluorescent labeling of slow-cycling cells, thereby facilitating flow sorting of viable cells for further characterization. Transgenic mice expressing histone H2B–GFP through a tetracycline-responsive regulatory element are crossed with mice expressing a tet repressor under the control of a basal epidermal layer‒specific promoter. Adult mice are fed tetracycline for several weeks, corresponding to the chase period in DNA labeling studies, and the cells that retain GFP are slow-cycling cells (Tumbar et al., 2004).

Whole-mount epidermal labeling methods have greatly facilitated the detection and quantitation of slow-cycling basal cells and also of basal cells that have initiated terminal differentiation. Intact sheets of human and mouse epidermis are separated from the underlying dermis enzymatically, labeled with appropriate antibodies, and then imaged by confocal microscopy, obviating the need to prepare histological sections (Braun et al., 2003; Jensen et al., 1999). BrdU label‒retaining cells can readily be detected in the bulge of mouse tail hair follicles using this method (Braun et al., 2003). In addition, the patterned distribution of cells that have initiated terminal differentiation in the basal layer and begun to express suprabasal keratins can be visualized in mouse and human epidermal whole mounts (Braun et al., 2003; Jensen et al., 1999). In mouse tail epidermis, the location of keratinocyte undergoing two different programs of terminal differentiation (scale and nonscale) can readily be observed (Gomez et al., 2013).

In summary, although the rate of cell division turns out not to be a robust marker of stem cells (Clevers and Watt, 2018; Jones et al., 2007), it is evident that the basal layer of the epidermis is heterogeneous, containing cells that differ in the rate at which they divide and whether or not they have initiated terminal differentiation.

## Heterogeneity of Prevalence of Genetic Mutations in the Epidermal Basal Layer

A different approach to exploring the heterogeneity of basal layer keratinocytes comes from studies of genetic mutations, whether induced by UV light or a manifestation of genetic skin disorders. Sun-exposed human skin contains clonal patches of p53-mutated keratinocytes that can be visualized by antibody staining of epidermal whole-mount preparations (Jensen et al., 1999; Jonason et al., 1996). The p53-mutant clones differ widely in the number of cells they contain, and their location can be interpreted as indicating that the founder cells are stem cells transit-amplifying cells, or suprabasal, differentiating cells (Jensen et al., 1999). By following the fate of clones in mice subjected to UVR, it is possible to discover how p53 mutation affects cell behavior (Klein et al., 2010). Such studies have led to the concept that mutations arising in terminally differentiating or transit-amplifying cells will be lost from the skin, whereas mutations arising in the stem cell compartment can confer characteristics that enhance their ability to populate the epidermis by outcompeting neighboring cells (Jensen et al., 1999). Nevertheless, the modeling data support the concept that the fate of any individual mutant proliferative cell is stochastic (Klein et al., 2010).

More recent studies have harnessed the power of deep sequencing to infer the clonality of epidermal cells through the detection of sunlight-induced somatic DNA mutations (Martincorena et al., 2015). There has been some debate about whether clone size can be fully accounted for by neutral drift, a process by which the emergence of mutant clones is through the genetic drift of mutant alleles that have neither a positive nor a negative effect on clone size (Martincorena et al., 2016, 2015; Simons, 2016a, 2016b). This has been resolved by sequencing larger areas (16 mm^2^ per donor) of cancer-prone skin than in earlier studies (Lynch et al., 2017). Skin spanning a range of ages of donors was analyzed because cancer incidence increases with age (Lynch et al., 2017). The conclusion from this study is that the distribution of clone sizes can be explained by a combination of neutral drift and stochastic nucleation of mutations at the boundary of expanding mutant clones that have a competitive advantage. Because *NOTCH* mutations are frequently observed in sun-exposed human epidermis, it seems plausible that they confer the observed competitive advantage, as in human esophageal epithelium (Colom et al., 2020).

Inherited skin disorders provide another means of examining the clonality of the human epidermis as a result of the phenomenon of genetic reversion, whereby inherited mutations are fully or partially corrected. Revertant mosaicism occurs in a variety of heritable disorders, including epidermolysis bullosa (EB). Up to 36% of EB patients with *COL17A1* mutations and 33% with *LAMB3* mutations exhibit revertant mosaicism (Lim et al., 2017). In cases where revertant skin has clinical characteristics that distinguish it from neighboring skin, the evidence of clonal heterogeneity is readily observable at the macroscopic level.

The conclusion from these studies is that within the epidermal basal layer, lineage relationships between cells can be inferred on the basis of the heterogeneity of genetic mutations. Different basal cells give rise to clones of differing sizes, size being dependent on elements of stochastic behavior (neutral drift), and survival advantages that are potentially conferred by specific mutations or locations within the tissue.

## In Vitro Stem Cell Assays and Markers

In parallel with the in vivo evidence for epidermal basal cell heterogeneity, techniques for culturing human keratinocytes have provided evidence for heterogeneity in vitro. The classic technique of plating human epidermal keratinocytes at clonal density on a feeder layer of 3T3 J2 cells (Rheinwald and Green, 1975) and subsequent demonstration that multilayered sheets formed by the merged clones could be used to treat patients (Gallico et al., 1984; O’Connor et al., 1981) established that stem cells can persist in culture. The original technique has been developed successfully over the years to provide methods for in vitro expansion of other epithelia for grafting and to combine cell and gene therapy for genetic skin disorders (De Luca et al., 2019; Hirsch et al., 2017).

Clonal growth of human keratinocytes has become a well-established method for stem cell quantitation. One assay involves disaggregating apparently uniform clones and then, on replating, scoring them as paraclones, meroclones, or holoclones on the basis of increasing ability to form large secondary clones (Barrandon and Green, 1987; Hirsch et al., 2017). Another involves scoring the size of individual clones and the proportion of differentiated cells within each clone after 14 days (Jones and Watt, 1993). The abortive clones are attributed to founder cells that have limited self-renewal ability (transit-amplifying cells), whereas large, actively growing clones are attributed to stem cells (Jones and Watt, 1993). The cell cycle time of clonogenic and abortive cells in these assays is the same, but the frequency of generating involucrin-positive cells differs (Jones and Watt, 1993). Involucrin is a cornified envelope precursor protein that is expressed in the upper spinous layers of healthy adult epidermis but in the lower spinous layers of hyperproliferative epidermis and in all suprabasal cells in epidermal cultures (Dover and Watt, 1987; Watt et al., 1987).

Using the simple clonal growth assay (rather than the holoclone assay) as a surrogate measure of the number of stem cells, it was possible to screen candidate cell surface markers of stem cells. The first reported marker was a high expression of β1 integrin extracellular matrix (ECM) receptors (Jones and Watt, 1993). Consistent with high integrin expression, clonogenic cells adhere more rapidly to ECM proteins than the cells that found abortive clones, providing a simple means of stem cell enrichment (Jones and Watt, 1993; Negri et al., 2019). Over the years, a range of cell surface markers of human epidermal stem cells have been reported, and it has been established that cells expressing those markers are found in clusters with a patterned distribution within the epidermal basal layer (Jones et al., 1995; Legg et al., 2003; Lowell et al., 2000).

## Defining Commitment in Cultured Human Keratinocytes

In addition to the search for markers of clonogenic epidermal stem cells, recent attention has focused on markers of commitment. These are genes that are expressed by basal cells that are no longer stem cells and are destined to differentiate but have yet to express suprabasal markers. The approach that we took was based on the observation that human keratinocytes undergo terminal differentiation when placed as a single-cell suspension in methylcellulose. We found that suspension-induced differentiation could be partially inhibited by β1-integrin ligation with antibodies or ECM proteins. However, after 4 hours in suspension, differentiation could not be inhibited, even though keratinocytes still expressed integrins and involucrin expression did not start to increase until 8 hours (Adams and Watt, 1989). We therefore hypothesized that 4 hours represented the time at which cells were committed to undergo terminal differentiation.

By integrating transcriptomic and proteomic data from keratinocytes held in suspension for different lengths of time, we identified a network of interacting protein phosphatases, including DUSP6, PPTC7, PTPN1, PTPN13, and PPP3CA, which are upregulated at 4 hours in suspension and promote differentiation by negatively regulating extracellular signal‒regulated kinase (ERK) MAPK and positively regulating activator protein 1 transcription factors (Mishra et al., 2017). The protein phosphatases act as an unstable commitment switch between two stable states (stem and differentiated). Whole-mount labeling of human epidermal sheets and reconstituted human epidermis shows that protein phosphatase expression is spatially regulated, with DUSP6 being most highly expressed in β1-integrin high basal cells (Mishra et al., 2017; Mobasseri et al., 2019). Lipidomic analysis has revealed an accumulation of numerous lipid species at different times during suspension-induced differentiation and identified candidate bioactive lipid subspecies as differentiation regulators (Vietri Rudan et al., 2020).

Although the suspension experiments provide temporal information about exit from the stem cell compartment, they are carried out on bulk cell populations. To examine the exit from the stem cell compartment at single-cell resolution, we measured ERK MAPK dynamics in cultured human keratinocytes using a fluorescence resonance energy transfer sensor for ERK and a fluorescent involucrin reporter (Hiratsuka et al., 2020). Stem cells were characterized as having high stable ERK activity, whereas involucrin-positive, differentiated cells had low stable basal ERK activity. We also identified two populations of keratinocytes with pulsatile ERK activity on a background of high or medium mean ERK activity. Transitions from Basal^hi^-Pulse^lo^ (stem) to Basal^hi^-Pulse^hi^ were reversible, whereas Basal^mid^-Pulse^hi^ cells were committed to undergo terminal differentiation. We found that basal ERK activity was regulated by DUSP10, whereas ERK pulses were regulated by DUSP6. This led us to speculate that DUSP6-mediated downregulation of ERK pulses promotes initiation of differentiation, whereas DUSP10-mediated downregulation of mean ERK activity promotes and stabilizes postcommitment differentiation (Hiratsuka et al., 2020).

In summary, experiments with cultured human keratinocytes have identified a number of markers for clonogenic cells, which are putative stem cells, and also potential regulators of exit from the stem cell compartment.

## Early Single-Cell Gene Expression Profiling

Given the limitations of relying on a candidate approach to identify stem cell markers, my laboratory explored whether we could find new markers by single-cell gene expression profiling. We generated cDNA libraries from single cultured human epidermal cells, designating them as basal cells if they expressed keratin (K) 5 and K14 and as stem or transit-amplifying cells depending on whether or not they expressed the stem cell markers DLL1 and melanoma-associated chondroitin sulfate proteoglycan (Jensen and Watt, 2006). The sensitivity of this early study was low, but we did identify 14 genes upregulated at least seven-fold in the stem cell libraries compared with those in the transit-amplifying cell libraries. This led to the identification of Kekkon/LIG1/LRIG1 as a stem cell marker (Jensen and Watt, 2006). LRIG1 is expressed in groups of basal cells in human IFE previously identified as enriched for stem cells (Jensen and Watt, 2006). Subsequent studies have found that LRIG1 is a marker of stem cells in a range of epithelia (Ji et al., 2021) and is also differentially expressed in subsets of mouse dermal fibroblasts (Gomez et al., 2013).

With some improvements in sensitivity, we went on to use single-cell global gene expression profiling not only to identify new makers of clonogenic keratinocytes but also to associate them with interacting signaling pathways, including ERK MAPK, which was already known to play a role in stem cell maintenance (Tan et al., 2013). We showed that basal cells fell into two clusters delineated by expression of DLL1. The DLL1+ cluster had elevated expression of genes associated with endocytosis, integrin-mediated adhesion, and receptor tyrosine kinase signaling. Overexpression of DLL1 alone or in combination with LRIG1 led to the upregulation of other genes in the DLL1+ cluster and resulted in enhanced ECM adhesion and caveolin-dependent EGFR endocytosis. This suggested that the stem cell marker genes were not independently regulated.

These early studies, although technically challenging and limited by sensitivity and by the number of cells analyzed, yielded new stem cell markers and suggested the power of analyzing global gene expression at single-cell resolution.

## Mapping Known Epidermal Cell Clusters by Single-Cell RNA Sequencing

Single-cell RNA-sequencing (scRNA-seq) studies received a major boost through rapidly advancing technology development, combined with the 2016 launch of the global initiative called The Human Cell Atlas (HCA) (Regev et al., 2017). The HCA is a collaborative effort to map all cell types in the body to gain new insights into normal human physiology and disease. The data flow from the HCA not only encompasses scRNA-seq data but also new methods for data analysis and new techniques to map the location of cells in tissues. Given the strength of the dermatology community, the variety of well-annotated skin diseases, and the accessibility of skin biopsies, it is not surprising that there have already been numerous important studies of skin cell heterogeneity since the HCA was launched (Cheng et al., 2018; Reynolds et al., 2021; Wang et al., 2020).

Whereas previous studies focused on cultured keratinocytes (Jensen and Watt, 2006; Tan et al., 2013), the new datasets include single-cell analysis of multiple cell types present in individual skin biopsies and encompass information about changes associated with inflammatory skin disease, cancer, and aging (He et al., 2020; Ji et al., 2020; Reynolds et al., 2021; Solé-Boldo et al., 2020; Zou et al., 2021). Because of the ease of data sharing, it is possible to analyze datasets from multiple laboratories, and the initial results are very encouraging, namely that the same cell populations are present in different datasets (Ascensión et al., 2021).

Similar to all experimental approaches, there are limitations to scRNA-seq. In the case of human skin, one of the key concerns is whether all the cell types present in a sample have been isolated and therefore whether their relative abundance in the datasets reflects their relative abundance in the tissue. To illustrate the type of approach that can be taken, Reynolds et al. (2021) used 200-mm-thick healthy skin mammoplasty samples. The upper 200 μm of skin was harvested using a dermatome and treated with dispase to separate epidermis from dermis, and then both fragments were separately digested in collagenase. This protocol enabled 34 distinct skin cell states in healthy adult skin to be distinguished, encompassing epidermis, fibroblasts, endothelial cells, and immune cells. Nevertheless, it is inevitable that not all cells would be isolated, and in particular, it is hard to achieve a good representation of granular epidermal cells given the abundance of desmosomes and adherens junctions (Cheng et al., 2018).

Reynolds et al. (2021) obtained over 500,000 scRNA-seq profiles, representing healthy adult skin, fetal skin, and lesional and nonlesional skin from patients with atopic dermatitis and psoriasis. We were particularly interested in whether the predictions of basal epidermal heterogeneity and the candidate stem cell markers from earlier studies could be validated in healthy adult epidermal keratinocytes. We therefore recently reanalyzed the scRNA-seq dataset comprising epidermis from healthy adult skin (Negri et al., unpublished data). The distinct epidermal cell types (basal, spinous, and granular) were annotated manually on the basis of known markers, and the most significantly expressed genes in each cluster and differential gene expression between distinct clusters were analyzed.

**Figure 1:**
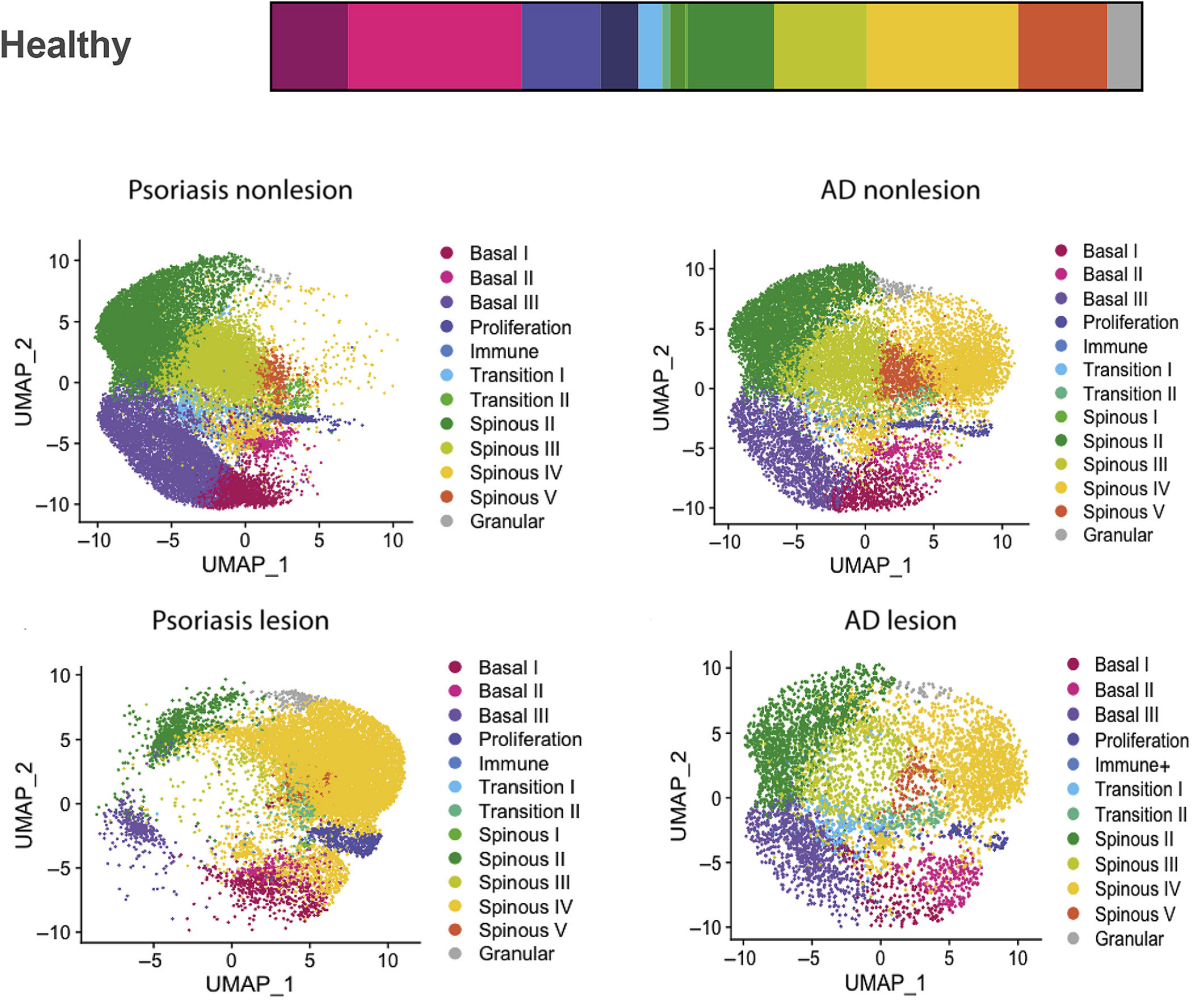
**Transcriptional heterogeneity of human epidermal keratinocytes in vivo.** Thirteen distinct cell states were identified in human interfollicular epidermis. The relative abundance of the states in healthy, nonlesional, and lesional atopic dermatitis and psoriasis is shown. Each dot represents one cell in the UMAP plots. Data are from Reynolds et al. (2021). AD, atopic dermatitis; UMAP, Uniform Manifold Approximation and Projection.

We were able to identify 13 distinct keratinocyte clusters (Figure 1). On the basis of the expression of K5 and K14, we could assign four clusters to the basal layer―basal I, II, and III, and proliferation―in good agreement with scRNA-seq of neonatal foreskin keratinocytes (Wang et al., 2020). The proliferation cluster expresses high levels of cycling cells, including *CDK1* and *MKI67*. The relative abundance of different integrin subunits differs across the basal clusters, with *ITGA3* and *ITGA6* most abundant in basal II and *ITGA2* and *ITGB1* most abundant in basal III. *DLL1* and another stem cell marker that had been characterized in culture, *CD46*, are most highly expressed in basal II. In culture, *CAV1* has been identified as a marker of *DLL1* high stem cells (Tan et al., 2013), but in keratinocytes isolated directly from skin, *CAV1* is most abundant in basal I.

As well as obtaining evidence for basal cell heterogeneity, we could identify two clusters as transition states on the basis of the expression of DUSP6 and DUSP10 (Mishra et al., 2017). *DUSP6* is primarily upregulated in transition I, whereas *DUSP10* is upregulated in transition I and II, in good agreement with the finding that *DUSP10* expression in suspension culture is upregulated for longer than that of *DUSP6* (Mishra et al., 2017). We could also distinguish five clusters as belonging to the spinous cell layers on the basis of the expression of *K1* and *K10*. A small cluster of granular cells was identified on the basis of *FLG* expression. Finally, we found a cluster of keratinocytes categorized as immune*,* expressing a mixture of basal (K5 and K14) and suprabasal (K10) markers and high levels of the macrophage migration inhibitory factor receptor CD74.

The scRNA-seq datasets of healthy epidermis not only confirm the heterogeneity of basal keratinocytes and validate previously reported stem cell markers (Reynolds et al., 2021) but also enable the identification of new stem cell markers. One example is COL17A1, which is upregulated in groups of basal keratinocytes that lie between rete ridges (projections of epidermis into the underlying dermis) (Wang et al., 2020). COL17A1 is thus expressed most highly in the same location as other stem cell markers, including *CD46* and *DLL1* (Jones et al., 1995; Legg et al., 2003; Lowell et al., 2000; Tan et al., 2013).

One area of ongoing investigation is whether the different basal cell subpopulations are equipotent, in terms of self-renewal and frequency of generating differentiated cells, or arranged in a hierarchy. A second is whether differential gene expression in basal I, II, and III is also reflected in different protein levels. This is of interest because markers such as ITGB1 and CD46 that are coexpressed by protein detection methods (flow cytometry and immunofluorescence staining) are differentially expressed at the transcript level and may reflect differences in protein turnover rates. For example, in the human breast epithelial cell line MCF10A, the protein half-lives of ITGB1, ITGA6, and CAV1 are approximately 15, 23, and 45 hours, respectively (Ly et al., 2018). Notwithstanding the differences in protein turnover, there do appear to be differences between cultured keratinocytes and keratinocytes isolated directly from the skin because in culture, DLL1+ basal cells express higher levels of ITGB1 than DLL1‒ basal cells (Tan et al., 2013), whereas that is not the case in the Reynolds et al. (2021) dataset.

## Changes in Keratinocytes associated with Psoriasis and Atopic Dermatitis

In addition to comparing different keratinocyte states in healthy adult skin, it is possible to use publicly available datasets to examine the changes in cell states associated with skin disease. We have used the Reynolds et al. (2021) dataset to analyze scRNA-seq of keratinocytes from patients with psoriasis and atopic dermatitis. Patients provided a 6-mm punch biopsy from lesional and nonlesional skin. The nonlesional samples were at least 2 cm away from lesional skin. In the dataset we analyzed, there were four patients with atopic dermatitis and three with psoriasis, of mixed sex and age (Reynolds et al., 2021).

The relative abundance of the 13 keratinocyte clusters found in healthy adult skin is altered in lesional and nonlesional epidermis from patients with psoriasis and atopic dermatitis (Figure 1), consistent with earlier studies (Kelemen et al., 2021; Luger et al., 2021). The marked expansion of the spinous layer clusters in the lesional epidermis (Figure 1) is consistent with the histology of the tissue, but it is interesting that the expansion is skewed to one cluster (spinous IV) in psoriasis but not in atopic dermatitis. The relative abundance of basal I, II, and III is also altered in psoriasis and atopic dermatitis (Figure 1), which may provide new insights into the nature of the stem cell compartment in hyperproliferative skin.

## Discussion

In recent years, the volume and quality of scRNA-seq datasets have increased considerably, providing new opportunities to explore the nature of epidermal stem cells and test the validity of in vitro experimental models. Overall, there is good agreement between the in vitro and in vivo datasets, and the changes in the relative abundance of different basal clusters suggest that there are changes in the stem cell compartment in psoriasis and atopic dermatitis. Nevertheless, there are many unanswered questions, including whether all skin cells are accounted for. The answer to this question is undoubtedly "no", given the under-representation of hair follicles and other adnexal structures in the existing datasets, the remarkable diversity of hair follicles, and the changes that take place during the hair growth cycle (Paus and Cotsarelis, 1999). Further considerations are whether the cell states within the human epidermis differ according to body site, hormone status, and ethnic background.

One of the current challenges―and opportunities―is to map the location of the different cell types revealed by scRNA-seq to specific spatial locations within the skin. Integration of high-dimensional multiomics approaches, including spatial transcriptomics and in situ sequencing, is already yielding interesting results (Ji et al., 2020). Furthermore, the skin lends itself to a variety of noninvasive imaging techniques that can provide temporal information about changes in response to the development and resolution of diseases (Heibel et al., 2020). Some techniques, such as optical coherence tomography, provide images that incorporate signals from the ECM, offering the tantalizing prospect that it will be possible to deduce changes in the stem cell compartment through changes in the niche.

## ORCIDs

Victor Augusti Negri: http://orcid.org/0000-0002-9188-1370

Fiona M. Watt: http://orcid.org/0000-0001-9151-5154

## Conflict of Interest

FMW receives research funding from Unilever and POLA.
